# Dynamic hyperinflation and intrinsic positive end-expiratory pressure in ARDS patients

**DOI:** 10.1186/s13054-019-2611-6

**Published:** 2019-11-27

**Authors:** Silvia Coppola, Alessio Caccioppola, Sara Froio, Erica Ferrari, Miriam Gotti, Paolo Formenti, Davide Chiumello

**Affiliations:** 1 Department of Anesthesia and Intensive Care, ASST Santi Paolo e Carlo, San Paolo University Hospital, Milan, Italy; 2 Department of Health Sciences, University of Milan, Milan, Italy; 3 Coordinated Research Center on Respiratory Failure, University of Milan, Milan, Italy; 4 SC Anestesia e Rianimazione, ASST Santi Paolo e Carlo, Via Di Rudinì, Milan, Italy

**Keywords:** Acute respiratory distress syndrome, Intrinsic positive end-expiratory pressure, Respiratory mechanics, Gas exchange

## Abstract

**Background:**

In ARDS patients, changes in respiratory mechanical properties and ventilatory settings can cause incomplete lung deflation at end-expiration. Both can promote dynamic hyperinflation and intrinsic positive end-expiratory pressure (PEEP). The aim of this study was to investigate, in a large population of ARDS patients, the presence of intrinsic PEEP, possible associated factors (patients’ characteristics and ventilator settings), and the effects of two different external PEEP levels on the intrinsic PEEP.

**Methods:**

We made a secondary analysis of published data. Patients were ventilated with a tidal volume of 6–8 mL/kg of predicted body weight, sedated, and paralyzed. After a recruitment maneuver, a PEEP trial was run at 5 and 15 cmH_2_O, and partitioned mechanics measurements were collected after 20 min of stabilization. Lung computed tomography scans were taken at 5 and 45 cmH_2_O. Patients were classified into two groups according to whether or not they had intrinsic PEEP at the end of an expiratory pause.

**Results:**

We enrolled 217 sedated, paralyzed patients: 87 (40%) had intrinsic PEEP with a median of 1.1 [1.0–2.3] cmH_2_O at 5 cmH_2_O of PEEP. The intrinsic PEEP significantly decreased with higher PEEP (1.1 [1.0–2.3] vs 0.6 [0.0–1.0] cmH_2_O; *p* < 0.001). The applied tidal volume was significantly lower (480 [430–540] vs 520 [445–600] mL at 5 cmH_2_O of PEEP; 480 [430–540] vs 510 [430–590] mL at 15 cmH_2_O) in patients with intrinsic PEEP, while the respiratory rate was significantly higher (18 [15–20] vs 15 [13–19] bpm at 5 cmH_2_O of PEEP; 18 [15–20] vs 15 [13–19] bpm at 15 cmH_2_O). At both PEEP levels, the total airway resistance and compliance of the respiratory system were not different in patients with and without intrinsic PEEP. The total lung gas volume and lung recruitability were also not different between patients with and without intrinsic PEEP (respectively 961 [701–1535] vs 973 [659–1433] mL and 15 [0–32] % vs 22 [0–36] %).

**Conclusions:**

In sedated, paralyzed ARDS patients without a known obstructive disease, the amount of intrinsic PEEP during lung-protective ventilation is negligible and does not influence respiratory mechanical properties.

## Background

In patients with acute respiratory failure, changes in respiratory mechanical properties (compliance and resistance) can cause incomplete deflation of the respiratory system at end-expiration, promoting dynamic hyperinflation [1, 2]. When this happens, the alveolar pressure at end-expiration can exceed atmospheric pressure or the positive end-expiratory pressure (PEEP) applied during mechanical ventilation, generating an occult or intrinsic PEEP [3, 4]. Thus, in mechanically ventilated patients, the total PEEP is the sum of the external PEEP provided by the ventilator and the intrinsic PEEP when present. Hyperinflation and intrinsic PEEP have been reported in up to 90% of the patients with acute exacerbation of chronic obstructive pulmonary disease (COPD). This is due to dynamic airway compression with an increase in lung compliance that can limit expiratory flow [5–7]. This limitation is seen when, at a given lung volume, expiratory flow cannot be increased by raising the expiratory driving pressure, which is the difference between the plateau and expiratory pressures at the Y-piece [8, 9].

In patients with acute respiratory distress syndrome (ARDS), intrinsic PEEP has been mainly reported only in the first days of mechanical ventilation with lower levels than in COPD patients [10]. In one of the first descriptions, Rossi et al. reported intrinsic PEEP in 10 of 14 patients, with values from 0.5 to 7.5 cmH_2_O [4]. Furthermore, when intrinsic PEEP is not taken into consideration, respiratory compliance can be significantly underestimated by about 48% [4]. Expiratory flow limitation is the main cause of intrinsic PEEP in 80–90% of ARDS patients [5, 11, 12]. The severity of the limitation closely relates with intrinsic PEEP and diminishes with the application of an external PEEP [11, 12].

In addition to the expiratory flow limitation, which mainly occurs during active breathing, in sedated ARDS patients, other mechanisms can be involved in the generation of intrinsic PEEP, such as mechanical ventilation per se or the ventilatory setting [13]. In ARDS patients, lung-protective ventilation with a low tidal volume and moderate/high respiratory rate to avoid or limit respiratory acidosis has been reported to promote the development of intrinsic PEEP and dynamic hyperinflation [14–16].

In up to 20% of ARDS patients, an airway closure (defined as the lack of communication between the proximal airways and alveoli) can be another factor responsible for the development of intrinsic PEEP [17–19].

The aim of this study was to evaluate, in a large population of mechanically ventilated ARDS patients, the presence and amount of intrinsic PEEP, any associated factors (patients’ characteristics and ventilator settings), and the effects of two different levels of external PEEP on intrinsic PEEP.

## Methods

### Study population

ARDS patients enrolled in seven previous studies [20–26] were analyzed. The institutional review board of each hospital approved each study, and written consent was obtained according to the regulations applicable in each institution.

Patients were retrospectively classified according to the Berlin criteria [27] for five studies [20, 22–25] and according to the American European Consensus Conference for two studies [21, 26]. The median time between diagnosis and enrollment was 2 days [1–5].

Exclusion criteria were age less than 16 years, pregnancy, asthma or chronic obstructive pulmonary disease (COPD) reported in the patient’s medical history, and hemodynamic instability.

Patients were classified according to the presence or absence of intrinsic PEEP at the end of an expiratory pause (3–5 s) when any total PEEP higher than an external PEEP of 5 cmH_2_O was detected.

### Study protocol

Patients were supine, orally intubated, sedated, and paralyzed, with a bolus of vecuronium [23, 24, 26] or rocuronium every 20 min [22] for the duration of the study, and thereafter according to clinical judgment. Patients were mechanically ventilated in volume control mode, and all measurements were recorded in volume control mode.

The tidal volume was set at 6–8 mL/kg of predicted body weight with a respiratory rate to maintain an arterial pH of 7.30–7.45. The inspiratory time was set at 33% of the total respiratory time using a square-wave inspiratory flow.

A recruitment maneuver was done to standardize lung volume history, in pressure-controlled ventilation at PEEP 5 cmH_2_O, with a plateau pressure of 45 cmH_2_O, I:E 1:1, and respiratory rate 10 breaths/min for 2 min [18]. Then, from a minimum of 20 min to a maximum of 60 min after the recruitment maneuver, the same tidal volume, respiratory rate, and oxygen fraction were applied and a PEEP trial (at PEEP 5 and 15 cmH_2_O) was done. At both PEEP levels, respiratory mechanics were measured, and blood gas analyses were done after 20 min. The external PEEP and total PEEP were measured at the end of the expiration of a regular breath and at the end of an expiratory pause (3–5 s). Intrinsic PEEP was defined as the total PEEP minus the external PEEP [4, 28].

### Respiratory mechanics measurements

Respiratory mechanics data were acquired with dedicated transducers. Flow at airway opening was measured with a heated pneumotachograph (Fleish 2, Lausanne, Switzerland). Airway pressure was measured proximally to the endotracheal tube with a pressure transducer (MPX 2010 DP. Motorola, Solna, Sweden). In all patients, a heat and moisture exchanger (DARTM, Convidien™, Adult-Pediatric) was placed between the endotracheal tube and the pneumotacograph.

A radio-opaque esophageal balloon (SmathCath Bicore, USA) was positioned in the lower third of the esophagus 35–40 cm from the nose. Esophageal pressure was measured by connecting the balloon, inflated with 1.0–1.5 mL of air, to a pressure transducer [29]. All traces were sampled at 100 Hz and processed on a dedicated data acquisition system (Colligo and Computo).

During inspiratory and expiratory pauses, the static airway and esophageal pressures were measured. The resulting physiological variables were computed according to standard formulas (see Additional file 1).

### Gas exchange

Arterial blood gases were analyzed. The alveolar dead space was computed measuring the end-tidal expired partial pressure of carbon dioxide with a CO_2_SMO monitor; the physiological dead space was computed according to the Enghoff modification of Bohr’s equation, measuring the mixed expired partial pressure of carbon dioxide with a CO_2_SMO monitor (Novametrix, Wallingford, UK) [30]. (see Additional file 1).

### Computer tomography scans

After the PEEP trial, patients were moved to the radiology department and two whole-lung computer tomography (CT) scans were taken, after a recruitment maneuver. During an end-expiratory pause at 5 cmH_2_O of PEEP and an inspiratory pause at 45 cmH_2_O of plateau pressure. Lung CT scans were taken using the following parameters: 110 mAs, tube voltage 120 kV, rotation time 0.5 s, collimation 128 × 0.6 mm, reconstruction matrix 512 × 512. In each CT slice, lung profiles were outlined manually and analyzed with a dedicated software package (Soft-E-Film, www.softefilm.eu). The total lung gas volume, weights, and amounts of the different compartments (not inflated, poorly inflated, well inflated, and over-inflated) were calculated as previously described [31].

Lung recruitability was computed as the ratio of the difference between not-inflated tissue at 5 cmH_2_O of PEEP and that at 45 cmH_2_O of plateau pressure to the total lung tissue at 5 cmH_2_O of PEEP [21].

### Statistical analysis

Data were not normally distributed, and non-parametric methods were applied. When not otherwise specified, categorical data are reported as frequencies and percentages, while continuous data are presented as medians [interquartile range]. Baseline characteristics of the patients with and without intrinsic PEEP were compared by the Mann-Whitney rank sum test.

Two-way repeated measures analysis of variance (ANOVA) followed by all pairwise multiple comparison procedures (Holm-Sidak method) was applied to investigate the effects of intrinsic and external PEEP on respiratory mechanics, CT data, and gas exchanges.

*p* values 0.05 or less were considered statistically significant. The statistical analysis was done with SigmaPlot 11.0 (Systat Software, San Jose, CA) and Prism 6.00 software (GraphPad, San Diego, CA, USA).

## Results

A total of 217 patients were studied in the early phase of ARDS, within 3 days [2–6] of onset. Their baseline clinical characteristics are reported in Table 1. Eighty-seven patients (40%) had intrinsic PEEP with a median of 1.1 [1.0–2.3] cmH_2_O at 5 cmH_2_O of PEEP. Among these 87, 3 (3%) had an intrinsic PEEP lower than 1 cmH_2_O, 71 (82%) between 1 and 3 cmH_2_O, and 13 (15%) higher than 3 cmH_2_O. Patients with intrinsic PEEP had a significantly higher body mass index and arterial carbon dioxide than those without intrinsic PEEP. The respiratory rate and minute ventilation were significantly higher (18 [16–21] vs 15 [12–20] breaths/min; 9.0 [7.6–11.6] vs 8.2 [6.8–9.7] L/min), while tidal volume was lower in patients with intrinsic PEEP (480 [420–550] vs 500 [450–600] mL) (Table 1; see Additional file 1: Figure S1).

**Table 1:** Baseline characteristics of the study population in relation to the presence of intrinsic PEEP

	Total population (*n* = 217)	Patients with intrinsic PEEP (*n* = 87)	Patients without intrinsic PEEP (*n* = 130)	*p*
Age (yr)	61 [48–73]	60 [48–61]	62 [48–73]	*0.69*
Female % (*n*)	32 (69)	28 (24)	35 (45)	*0.30*
Height (cm)	170 [162–177]	170 [163–180]	170 [162–175]	*0.34*
BMI (kg/m^2^)	25 [22–29]	27 [23–29]	24 [22–28]	***0.004***
Intensive care unit stay (days)	18 [18–26]	19 [10–28]	17 [10–25]	*0.58*
SAPS II score	40 [32–53]	41 [33–50]	40 [32–53]	*0.82*
Hospital mortality % (*n*)	46 (99)	46 (47)	40 (52)	***0.05***
PaO_2_/FiO_2_	173 [132–224]	159 [119–193]	188 [142–225]	***0.008***
PaCO_2_ (mmHg)	42 [37–49]	44 [39–51	41 [36–48]	***0.005***
ARDS category % (*n*)				*0.09*
• Mild	18 (40)	16 (14)	15 (20)	
• Moderate	62 (135)	58 (50)	65 (85)	
• Severe	20 (42)	26 (23)	20 (25)	
Cause of ARDS % (*n*)				*0.78*
• Pulmonary	57 (123)	55 (48)	58 (75)	
• Extrapulmonary	43 (94)	45 (39)	42 (55)	
Respiratory rate (breath/min)	16 [14–20]	18 [16–21]	15 [12–20]	***< 0.0001***
Tidal volume (mL)	500 [430–586]	480 [420–550]	500 [450–600]	***0.03***
Tidal volume/body weight (mL/kg)	7.8 [6.8–8.8]	7.6 [6.7–8.3]	7.9 [6.9–9.4]	***0.0095***
Clinical PEEP (cmH_2_O)	10 [10–12]	10 [10–12]	10 [10–12]	*0.24*
Minute ventilation (L min^−1^)	8.5 [7.2–10.2]	9.0 [7.6–11-6]	8.2 [6.8–9.7]	***0.001***
Respiratory system compliance (mL cmH_2_O^−1^)	38 [32–50]	37 [30–45]	41 [33–51]	*0.11*

Student’s *t* test or Mann-Whitney rank sum test, as appropriate, were used to analyze continuous variables and the chi-square or Fisher’s exact test, as appropriate, for non-continuous variables. Data are expressed as mean (standard deviation) or median [I.Q. range] as appropriate

*N* sample size, *yr* years, *BMI* body mass index, *ARDS* acute respiratory distress syndrome, *SAPS* Simplified Acute Physiology Score

Italics: for all the statistical data

Bold italics: for statistical significant data

### Respiratory mechanics at different PEEP

The intrinsic PEEP significantly on raising PEEP from 5 to 15 cmH_2_O (1.1 [1.0–2.3] vs 0.6 [0.0–1.0] cmH_2_O; *p* < 0.001).

**Fig. 1:**
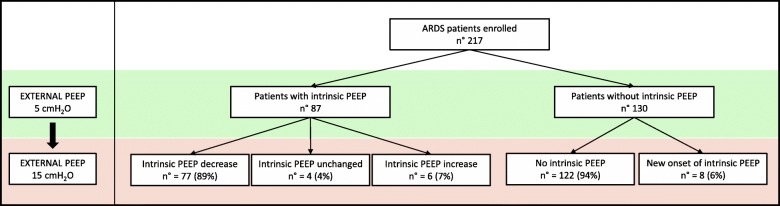
The effect of external PEEP in patients with and without intrinsic PEEP

Among these patients with intrinsic PEEP at 5 cmH_2_O, when external PEEP was raised, 89% presented a decrease in intrinsic PEEP with a median of − 1 [− 2.00 to − 0.96], 7% showed an increase (0.6 [0.17–1.25] cmH_2_O), and only 4% had no change. Eight of the patients without intrinsic PEEP (6%) developed a small amount of intrinsic PEEP while the remaining 94% did not (Fig. 1).

At both 5 and 15 cmH_2_O of PEEP, the applied tidal volume was significantly lower in patients with intrinsic PEEP (480 [430–540] vs 520 [445–600] mL at 5 cmH_2_O; 480 [430–540] vs 510 [430–590] at 15 cmH_2_O) while the respiratory rate was significantly higher (18 [15–20] vs 15 [13–19] bpm at 5 cmH_2_O; 18 [15–20] vs 15 [13–19] bpm at 15 cmH_2_O) (Table 2).

**Table 2:** Respiratory mechanics at different PEEP in patients with and without intrinsic PEEP

Parameter	Patients with intrinsic PEEP (*n* = 87)	Patients without intrinsic PEEP (*n* = 130)	*P*_PEEPi_	*P*_PEEP_	*P*_PEEPix PEEP_
Tidal volume (mL)			***0.012***	*0.357*	*0.874*
5 cmH_2_O	480 [430–540]	520 [445–600]			
15 cmH_2_O	480 [430–540]	510 [430–590]			
Respiratory rate (breath/min)			***< 0.001***	*0.992*	*0.954*
5 cmH_2_O	18 [15–20]	15 [13–19]			
15 cmH_2_O	18 [15–20]	15 [13–19]			
Minute ventilation (L/min)			***0.007***	0.559	***0.048^***
5 cmH_2_O	8.6 [7.6–10.2]	8.0 [6.9–9.4]			
15 cmH_2_O	8.6 [7.3–10.7]	7.9 [6.8–9.4]			
Plateau pressure (cmH_2_O)			*0.726*	***< 0.001***	*0.061*
5 cmH_2_O	18.4 [16.3–21.4]	18.0 [15.5–20.2]			
15 cmH_2_O	27.8 [24.9–30.0]	28.0 [25.4–30.0]			
Expiratory time (s)			***0.002***	*0.884*	*0.774*
5 cmH_2_O	2.36 [1.99–2.93]	2.80 [2.12–3.43]			
15 cmH_2_O	2.31 [1.92–2.92]	2.80 [2.12–3.40]			
Total resistance (cmH_2_O s L^−1^)			*0.246*	*0.679*	*0.286*
5 cmH_2_O	13 [11–20]	14 [11–18]			
15 cmH_2_O	13 [10–18]	15 [12–19]			
Respiratory system compliance (mL cmH_2_O^−1^)			*0.170*	***0.023***	*0.698*
5 cmH_2_O	43 [34–53]	41 [31–52]			
15 cmH_2_O	41 [30–51]	38 [33–49]			
Lung compliance (mL cmH_2_O^−1^)			***0.004***	*0.088*	*0.250*
5 cmH_2_O	57 [44–78]	57 [43–71]			
15 cmH_2_O	57 [44–72]	54 [41–66]			
Chest wall compliance (mL cmH_2_O^−1^)			*0.981*	*0.564*	*0.388*
5 cmH_2_O	174 [125–272]	178 [116–269]			
15 cmH_2_O	160 [103–272]	158 [111–238]			
Time constant (s)			*0.192*	*0.549*	*0.554*
5 cmH_2_O	0.65 [0.43–0.79]	0.63 [0.45–1.00]			
15 cmH_2_O	0.56 [0.36–0.77]	0.57 [0.44–0.73]			

Data are expressed as median [I.Q. range] as appropriate

Two-way repeated measures analysis of variance (ANOVA) followed by all pairwise multiple comparison procedures (Holm-Sidak method) were used for analysis

*n* sample size, *PEEP* positive-end expiratory pressure

^^^*p* < 0.05: minute ventilation is higher in patients with intrinsic PEEP than those without intrinsic PEEP at PEEP 15. There are no other significant interactions

Italics: for all the statistical data

Bold italics: for statistical significant data

Lung compliance was significantly higher in patients with intrinsic PEEP (*p* = 0.004) while the airway plateau pressure and driving pressure were not different (Table 2 and Additional file 1: Table S1).

Expiratory time was lower than 3 time constants in 56 patients (26%) out of the whole population; this happened in 30 (34%) patients with and 26 (20%) without intrinsic PEEP. The expiratory time was lower in patients with intrinsic PEEP at both PEEP levels while total airway resistance and compliance of the respiratory system were not different in patients with and without intrinsic PEEP (Table 2).

### Computer tomography

Table 3 shows the CT data at 5 and 45 cmH_2_O of airway pressure. The total lung gas volume was no different in the two groups at the two pressures. In both groups, the percentages of not inflated and poorly inflated tissue were similarly reduced while the well-inflated tissue increased from 5 to 45 cmH_2_O of airway pressure.

**Table 3:** Computed tomography data in patients with and without intrinsic PEEP

	Patients with intrinsic PEEP (*n* = 87)	Patients without intrinsic PEEP (*n* = 130)	*P*_PEEPi_	*P*_PEEP_	*P*_PEEPix PEEP_
Total weight (g)			*0.72*	*N.A.*	*N.A.*
5 cmH_2_O	1366 [1151–1666]	1411 [1172–1674]			
Total gas volume (mL)			*0.79*	*N.A.*	*N.A.*
5 cmH_2_O	961 [701–1535]	973 [659–1433]			
Non-inflated tissue (%)			*0.242*	***< 0.001***	*0.301*
5 cmH_2_O	39 [26–53]	43 [33–55]			
45 cmH_2_O	25 [18–35]	27 [15–38]			
Poorly inflated tissue (%)			*0.815*	***< 0.001***	*0.490*
5 cmH_2_O	30 [23–39]	28 [21–40]			
45 cmH_2_O	21 [18–33]	25 [19–35]			
Well-inflated tissue (%)			*0.203*	***< 0.001***	*0.888*
5 cmH_2_O	26 [15–40]	25 [14–34]			
45 cmH_2_O	43 [37–56]	43 [34–55]			
Over-inflated tissue (%)			*0.814*	***< 0.001***	*0.149*
5 cmH_2_O	0.01 [0–0.13]	0.01 [0–0.11]			
45 cmH_2_O	0.7 [0.21–4.17]	1.49 [0.36–4.60]			

Data are expressed as median [I.Q. range] as appropriate

Two-way repeated measures analysis of variance (ANOVA) followed by all pairwise multiple comparison procedures (Holm-Sidak method) was used for analysis

*p* < 0.05 raising airway pressure from 5 to 45 cmH_2_O

There are no significant interactions

*n* sample size, *PEEP* positive end-expiratory pressure

Italics: for all the statistical data

Bold italics: for statistical significant data

### Lung recruitability and gas exchange

Lung recruitability was not different among patients with and without intrinsic PEEP (15 [0–32] % vs 22 [0–36] %).

Pulmonary gas exchange data are presented in Table 4. PaO_2_ was similar between patients with and without intrinsic PEEP at 5 cmH_2_O (71 [64–83] mmHg vs 73 [63–93]) and similarly increased at 15 cmH_2_O (94 [75–124] mmHg vs 99 [80–123] mmHg). PaCO_2_ and physiological and alveolar dead space did not differ between the two groups (Table 4 and Additional file 1: Table S2).

**Table 4:** Gas exchange at different levels of PEEP in patients with and without intrinsic PEEP

Parameter	Patients with intrinsic PEEP (*n* = 87)	Patients without intrinsic PEEP (*n* = 130)	*P*_PEEPi_	*P*_PEEP_	*P*_PEEPix PEEP_
PaO_2_ (mmHg)			0.761	***< 0.001***	0.939
5 cmH_2_O	71 [64–83]	73 [63–93]			
15 cmH_2_O	94 [75–124]	99 [80–123]			
PaO_2_/FiO_2_			*0.118*	***< 0.001***	0.957
5 cmH_2_O	138 [100–183]	151 [119–185]			
15 cmH_2_O	194 [144–252]	200 [159–259]			
PaCO_2_ (mmHg)			*0.111*	0.518	0.787
5 cmH_2_O	44 [40–52]	44 [38–51]			
15 cmH_2_O	44 [41–52]	38 [22–42]			
Alveolar dead space (%)			*0.279*	*0.345*	*0.425*
5 cmH_2_O	20 [12–26]	23 [13–30]			
15 cmH_2_O	19 [13–27]	21 [11–29]			
Alveolar dead space (mL)			*0.186*	***0.010***	*0.107*
5 cmH_2_O	105 [66–137]	111 [74–153]			
15 cmH_2_O	98 [68–131]	105 [62–140]			

Data are expressed as median [I.Q. range] as appropriate

Two-way repeated measures analysis of variance (ANOVA) followed by all pairwise multiple comparison procedures (Holm-Sidak method) was used for analysis

*p* < 0.05 raising end-expiratory pressure from 5 to 15 cmH_2_O

There are no significant interactions

*n* sample size, *PEEP* positive end-expiratory pressure

Italics: for all the statistical data

Bold italics: for statistical significant data

## Discussion

In this population of ARDS patients evaluated in the early phase of the disease, (1) intrinsic PEEP was detected in 40%, with a median of 1.1 [1.0–2.3] cmH_2_O at 5 cmH_2_O of PEEP; (2) patients with intrinsic PEEP had a ventilatory setting with a lower tidal volume and higher respiratory rate, higher lung compliance, and similar time constant, lung gas volume, and gas exchange compared to patients without intrinsic PEEP; and (3) when the external PEEP was raised from 5 to 15 cmH_2_O, intrinsic PEEP decreased significantly.

The first descriptions of dynamic hyperinflation and intrinsic PEEP in COPD patients were reported by Pepe and Marini [3]. In the presumed pathophysiological mechanism is the failure for the lung units to empty their volume and to reach their relaxation volume before a new inspiration is initiated, mainly due to the expiratory flow limitated by airway collapse [3, 4, 10, 32, 33].

In ARDS patients, dynamic hyperinflation and intrinsic PEEP can be generated by the expiratory flow limitation and by the combination of short expiratory time and high respiratory rate when the lungs do not have enough time to reach their equilibrium volume during passive deflation [2, 4, 32, 34]. Dynamic hyperinflation and intrinsic PEEP can have several negative effects on the respiratory system and on hemodynamics. They may increase the work of breathing and the lung gas volume and reduce the preload and cardiac output [3, 16].

At the bedside, intrinsic PEEP can be suspected whenever the expiratory gas flow is abruptly interrupted by the next inflation of the ventilator. In mechanically ventilated patients, dynamic hyperinflation can be quantified as the amount of intrinsic PEEP computed as a positive pressure higher than the PEEP set on the ventilator during a prolonged expiratory pause in the absence of any expiratory flow limitation. Otherwise dynamic hyperinflation can be quantified as the amount of total PEEP not explained by the external PEEP set on the ventilator: this may happen when an increase in the external PEEP does not cause a proportional increase in the total PEEP, when there is a limitation of expiratory flow. This method is accurate only when patients are well sedated with or without paralysis [3, 4, 32, 33]. The ARDS patients in the present study were sedated and paralyzed. They were ventilated with low tidal volume and a clinical, not preselected, respiratory rate and expiratory time to avoid respiratory acidosis, reflecting a common ventilatory strategy of ARDS patients in intensive care [35].

### Respiratory mechanics and body mass index

Regarding respiratory mechanics, only lung compliance was significantly different but not to any clinically relevant extent. In patients without muscle activity, deflation of the respiratory system is completely passive and the entire force for exhalation derives from the elastic potential energy stored in the respiratory system during the inspiration [36]. Taking into account the equation of motion of the respiratory system, the time required for complete exhalation depends on the product of total resistance and compliance, which is the time constant of the respiratory system [37]. Assuming a homogeneous lung compartment model with only one time constant, where the expiratory time equals the product of resistance for compliance, the volume that will remain in the respiratory system should be approximately 37% of the inspired volume [36]. Three time constants are required to reach a 95% volume exhalation [38, 39]. Thus, for a given applied tidal volume, if there is a significant increase in compliance or resistance, it will take longer to reach the resting volume. ARDS patients, characterized by reduced compliance [1], should be theoretically protected from developing dynamic hyperinflation [28, 39].

On the other hand, an increase in resistance can promote the development of dynamic hyperinflation. In the present study, the product of compliance and resistance was not different (i.e., time constant) in the two groups, suggesting the respiratory mechanics have no role in promoting intrinsic PEEP.

Morbidly obese patients compared with normal-weight patients, especially in a supine position and during general anesthesia, can present a combination of factors, such as expiratory flow limitation and a larger amount of peripheral airway closure volume [40, 41], that can result in intrinsic PEEP. Grieco et al., with a low-flow P-V curve technique, showed that 22% of obese patients under general anesthesia presented airway closure with a median airway opening pressure of 9 cmH_2_O [42].

Similar data were reported in ARDS patients by Yonis et al. who found a significantly higher airway opening pressure in patients with a higher body mass index [18].

As expected, in the present study, the patients with intrinsic PEEP had a significantly higher body mass index, even if lower than 30 kg/m^2^, than patients without intrinsic PEEP. However, since in the present study we did not have a P-V curve loop, we could not assess the possible presence of airway closure which has been reported between 25 and 50% of ARDS patients [17, 19].

### Ventilatory settings

In mechanically ventilated patients, the increased expiratory airway resistance caused by the endotracheal tube and by the expiratory valve of the mechanical ventilator can promote dynamic hyperinflation [43]. In this study, although we did not record the sizes of the endotracheal tubes employed, we can assume there were no differences between the two groups because the percentages of males and females were similar. Our policy is to use larger endotracheal tubes in males than in females.

As regards the expiratory valve, we did not use the same mechanical ventilators in all patients; however, our latest-generation intensive care mechanical ventilators are able to limit any expiratory resistance as much as possible.

Under passive conditions, the short expiratory time, the high respiratory rate, and the minute ventilation promote dynamic hyperinflation [2, 44]. In ARDS patients, low tidal volume, which reduces stress and strain, is widely recommended in order to reduce ventilator-induced lung injury (VILI) [35]. However, to avoid respiratory acidosis, the respiratory rate is commonly raised to maintain adequate minute ventilation and carbon dioxide clearance. Richard et al. found that raising the respiratory rate from 17 to 30 breaths/min significantly increased intrinsic PEEP, from 1.3 to 3.9 cmH_2_O, maintaining the same low tidal volume (6 mL/kg of predicted body weight) [14]. However, in our study, the respiratory rate and minute ventilation were increased simultaneously, and they can promote dynamic hyperinflation [44], although our intrinsic PEEP was lower. For a similar minute ventilation (12 L/min), obtained with a different combination of low/high tidal volume and low/high respiratory rate, De Durante et al. found a progressive increase in intrinsic PEEP from 1.4 to 5.8 cmH_2_O with an increase in respiratory rate from 14 to 34 breaths/min [15].

In line with previous data, we found that patients with intrinsic PEEP were ventilated with higher respiratory rate and lower expiratory time than those without. Furthermore, the amount of intrinsic PEEP found in our study should approximately correspond to what was reported by Richard et al. for similar respiratory rates [14].

### PEEP response

Whenever an external PEEP is changed, the resulting new intrinsic PEEP is computed as the difference between the total PEEP measured during an expiratory pause and the applied by the ventilator [5, 28]. In ARDS patients, the PEEP is commonly used not only to improve gas exchange [45] but also to reduce lung inhomogeneity [20] and optimize recruitability [46, 47]. In a mixed population of COPD patients and patients with acute respiratory failure, when there was expiratory flow limitation, the higher external PEEP significantly reduced the intrinsic PEEP, which was “absorbed” in the total PEEP. In contrast, in patients without expiratory flow limitation, the intrinsic PEEP did not change and the total PEEP could be increased [12, 48].

Many studies have proposed raising the PEEP to detect any expiratory flow limitation: an external PEEP higher than the intrinsic PEEP can reduce the expiratory flow on the flow-volume loop [49, 50]. In the present study, on raising the applied PEEP from 5 to 15 cmH_2_O, intrinsic PEEP was significantly reduced in 89% of the patients and increased only in 4%; this might be due to airway closure and flow limitation at PEEP 5 and an airway opening pressure between 5 and 15 cmH_2_O that absorbs intrinsic PEEP. However, this value is clinically not important, suggesting expiratory flow limitation has no substantial role. In addition, the response in oxygenation was no different among patients with or without intrinsic PEEP. In fact, as Koutsoukou et al. showed arterial oxygenation significantly increased with the rise in external PEEP independently from the amount of intrinsic PEEP [12].

The two groups presented similar amounts of not consolidated tissue, lung gas volume, and lung recruitability, while the PaO_2_/FiO_2_ ratio was worse and the mortality rate was slightly higher in patients with intrinsic PEEP. Although in ARDS patients PaO_2_/FiO_2_ is commonly used in clinical practice to assess the severity and the response to ventilation and therapy, it is not a “research” indicator of the impairment of oxygenation (i.e., alveolar shunt). In fact, several extrapulmonary factors such as the concentration of hemoglobin, cardiac output, and the arterial-venous oxygen content can significantly affect PaO_2_/FiO_2_, independently from the severity of lung disease.

### Limitations

Limitations of this study are as follows: (1) the absence of data on airway resistance, because we only computed the total resistance including the endotracheal tubes; (2) the absence of any diagnosis of the expiratory flow limitation on the flow-volume loop or measuring the end-expiratory lung volume; and (3) the lack of measurements of intrinsic PEEP at zero PEEP.

## Conclusions

In conclusion, in sedated, paralyzed ARDS patients without a known obstructive disease, the amount of intrinsic PEEP during lung-protective ventilation is negligible and does not influence gas exchange or the respiratory mechanics in terms of response to external PEEP and lung recruitability.

## Supplementary information

**Additional file 1.** Calculations of physiological variables: *formulas used to calculate all physiological variables;* Respiratory mechanics variables: *data about driving pressure, transpulmonary driving pressure, elastance-derived transpulmonary pressure and end-expiratory transpulmonary pressure;* Dead space: *data about alveolar, physiological and anatomic dead space expressed both as volume and percentage;* Comparison between Tidal Volume and Respiratory Rate in patients with and without intrinsic PEEP: *boxplots describe the comparison between Tidal Volume in patients with and without intrinsic PEEP at 5 cmH*_*2*_*O on the left panel; the comparison between Respiratory Rate in patients with and without intrinsic PEEP at 5 cmH*_*2*_*O on the right panel.*

## Data Availability

The datasets used and analyzed in the study are available from the corresponding author on reasonable request*.*

## Abbreviations

ARDS: Acute respiratory distress syndrome
C_CW_: Chest wall compliance
C_L_: Lung compliance
COPD: Chronic obstructive pulmonary disease
C_RS_: Respiratory system compliance
CT: Computed tomography
DP: Driving pressure
PEEP: Positive end-expiratory pressure
*P*_Plat_: Plateau pressure, end-inspiratory airway pressure at end-inspiratory pause
*R*_max, rs_: Total resistance
VILI: Ventilator-induced lung injury
*V*t: Tidal volume
