# Supplementary material for: Dynamic hyperinflation and intrinsic positive end-expiratory pressure in ARDS patients
Source: Crit Care. 2019 Nov 27;23:375. doi: 10.1186/s13054-019-2611-6 (PMC6880369; doi:10.1186/s13054-019-2611-6)
Supplement: Supplementary file 1 — Additional file 1. Calculations of physiological variables: formulas used to calculate all physiological variables; Respiratory mechanics variables: data about driving pressure, transpulmonary driving pressure, elastance-derived transpulmonary pressure and end-expiratory transpulmonary pressure; Dead space: data about alveolar, physiological and anatomic dead space expressed both as volume and percentage; Comparison between Tidal Volume and Respiratory Rate in patients with and without intrinsic PEEP: boxplots describe the comparison between Tidal Volume in patients with and without intrinsic PEEP at 5 cmH2O on the left panel; the comparison between Respiratory Rate in patients with and without intrinsic PEEP at 5 cmH2O on the right panel. [file 13054_2019_2611_MOESM1_ESM.pdf]

Dynamic hyperinflation and intrinsic positive end-expiratory pressure in ARDS patients

Coppola Silvia, Caccioppola Alessio, Froio Sara, Ferrari Erica, Gotti Miriam, Formenti Paolo, Chiumello Davide

SUPPLEMENTARY MATERIAL

Calculations of physiological variables

The following equations were used for the computation of physiological respiratory variables.

Driving pressure = End inspiratory airways pressure – End expiratory airways pressure at PEEP

Respiratory system compliance = Tidal volume / (End inspiratory airways pressure – End expiratory airways pressure at PEEP)

Chest wall compliance = Tidal volume / (End inspiratory esophageal pressure – End expiratory esophageal pressure)

1/Lung compliance = 1/Respiratory system compliance – 1/Chest wall compliance

Total resistance = (Maximum value of airway pressure during inspiration – End inspiratory airways pressure) / Inspiratory flow

Time constant of the respiratory system = Total resistance · Respiratory system compliance

Transpulmonary driving pressure = Driving pressure – (End inspiratory esophageal pressure – End expiratory esophageal pressure)

Elastance-derived transpulmonary pressure = End inspiratory airways pressure · (Lung elastance / Respiratory system elastance)

End-expiratory transpulmonary pressure = End expiratory airways pressure – End expiratory esophageal pressure at PEEP

Physiological dead space = (Arterial partial pressure of carbon dioxide - Mixed expired partial pressure of carbon dioxide*) / Arterial partial pressure of carbon dioxide

**This variable* ($PECO_2$) *was automatically computed by the* CO_2*SMO monitor (Novametrix, Wallingford, CT)*

Alveolar dead space = (Arterial partial pressure of carbon dioxide – End tidal expired partial pressure of carbon dioxide*) / Arterial partial pressure of carbon dioxide

**This variable* ($PETCO_2$) *was automatically computed by the* CO_2*SMO monitor (Novametrix, Wallingford, CT)*

Anatomical dead space = Physiological dead space - Alveolar dead space

Table S1 Respiratory mechanics variables

Parameter	Patients with intrinsic PEEP (n = 87)	Patients without intrinsic PEEP (n = 130)	P_{PEEPi}	P_{PEEP}	$P_{PEEPi x PEEP}$
**Driving pressure (cmH_2O)**			*0.432*	*0.052*	*0.549*
5 cmH_2O	12.0 [9.3-14.7]	13.3 [10.5-15.3]			
15 cmH_2O	12.3 [10.0-15.0]	13.5 [11.0-15.9]			
**Transpulmonary driving pressure (cmH_2O)**			*0.187*	*0.301*	*0.530*
5 cmH_2O	9.8 [6.8-12.1]	11.0 [8.2-14.1]			
15 cmH_2O	9.5 [7.8-12.8]	10.9 [8.8-15.0]			
**Elastance-derived transpulmonary pressure (cmH_2O)**			*0.862*	***<0.001***	*0.159*
5 cmH_2O	15.0 [12.0-19.0]	15.0 [12.0-19.0]			
15 cmH_2O	20.0 [17.0-23.0]	21.0 [18.0-24.0]			
**End-expiratory transpulmonary pressure (cmH_2O)**			*0.809*	***<0.001***	*0.067*
5 cmH_2O	-7.4 [-11.0- -3.6]	- 8.0 [-11.4 - -5.4]			
15 cmH_2O	-2.1 [-4.2 – 2.0]	-1.3 [-3.8 – 1.9]			

Data are expressed as median [I.Q. range] as appropriate. n, sample size.

Two Way Repeated Measures ANalysis Of VAriance followed by All Pairwise Multiple Comparison Procedures (Holm-Sidak method) have been used for analysis, as appropriate.

$p<0.05$ when PEEP increases from 5 to 15 cmH_2O the variable significantly changes independently from the presence of intrinsic PEEP.

There are no statistically significant interactions.

Table S2. Dead space

Parameter	Patients with intrinsic PEEP (n = 39)	Patients without intrinsic PEEP (n = 77)	P_{PEEPi}	P_{PEEP}	$P_{PEEPix\ PEEP}$
**Alveolar dead space (%)**			*0.723*	*0.114*	*0.168*
5 cmH2O	22 [18-27]	24 [15-30]			
15 cmH2O	20 [17-28]	21 [14-29]			
**Alveolar dead space (mL)**			*0.712*	*0.060*	*0.282*
5 cmH2O	114.1 [86.6-148.7]	112.4 [76.5-153.3]			
15 cmH2O	107.3 [79.0-130.1]	108.8 [74.7-139.7]			
**Physiological dead space (%)**			*0.479*	***<0.001***	*0.601*
5 cmH2O	60 [56-70]	62 [52-69]			
15 cmH2O	62 [58-72]	63 [54-71]			
**Physiological dead space (mL)**			*0.861*	***0.039***	*0.587*
5 cmH2O	318.6 [288.6-352.2]	317.8 [272.1-357.5]			
15 cmH2O	325.9 [284.6-364.3]	318.9 [281.7-359.2]			
**Anatomic dead space (%)**			*0.485*	***<0.001***	*0.353*
5 cmH2O	37 [33-45]	36 [33-41]			
15 cmH2O	38 [35-50]	41 [37-45]			
**Anatomic dead space (mL)**			*0.203*	***<0.001***	*0.180*
5 cmH2O	198.0 [162.9-231.4]	196.1 [165.1-221.5]			
15 cmH2O	206.3 [169.8-258.7]	206.7 [182.8-245.5]			

Data are expressed as median [I.Q. range] as appropriate. n, sample size; PEEP, Positive End Expiratory Pressure. Two Way Repeated Measures ANalysis Of VAriance followed by All Pairwise Multiple Comparison Procedures (Holm-Sidak method) have been used for analysis, as appropriate.

$p<0.05$ when PEEP increases from 5 to 15 cmH_2O the dead space significantly increases independently from the presence of intrinsic PEEP.

There are no statistically significant interactions.

The physiological dead space was automatically computed by the CO2SMO monitor (Novametrix, Wallingford, CT), and was obtained in 39 (45%) and 77 (59%) patients, respectively in the group with and without intrinsic PEEP, as only in these patients mixed expired partial pressure of carbon dioxide (PECO2) was measured.

Figure S1

Comparison between Tidal Volume and Respiratory Rate in patients with and without intrinsic PEEP.

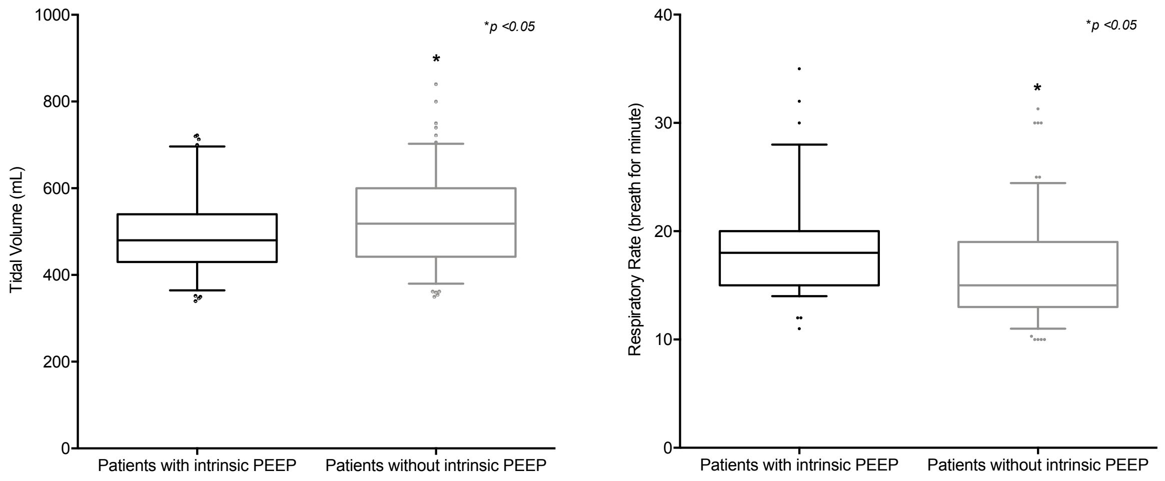

Boxplots describe the comparison between Tidal Volume in patients with and without intrinsic PEEP at 5 cmH_2O on the left panel; the comparison between Respiratory Rate in patients with and without intrinsic PEEP at 5 cmH_2O on the right panel.

Mann-Whitney Rank Sum Test, $p<0.05$. The median is represented by horizontal line in the box. The upper line of the box represents the upper quartile (75%), the lower line of the box represents the lower quartile (25%).
